# TMEM52B Isoforms P18 and P20 Differentially Promote the Oncogenesis and Metastasis of Nasopharyngeal Carcinoma

**DOI:** 10.1002/advs.202402457

**Published:** 2024-06-28

**Authors:** Yuqi Zhu, Yanxin Lu, Chunhua Xu, Yuqian Huang, Ziyi Yu, Tongyu Wang, Longyi Mao, Ximian Liao, Shi Li, Wanqing Zhang, Feng Zhou, Kaiqing Liu, Yu Zhang, Wei Yang, Shasha Min, Yaqin Deng, Zaixing Wang, Xiaoqin Fan, Guohui Nie, Xina Xie, Zesong Li

**Affiliations:** ^1^ Guangdong Provincial Key Laboratory of Systems Biology and Synthetic Biology for Urogenital Tumors Shenzhen Key Laboratory of Genitourinary Tumor Department of Urology Shenzhen Institute of Translational Medicine The First Affiliated Hospital of Shenzhen University Shenzhen Second People's Hospital Shenzhen 518000 China; ^2^ Guangdong Key Laboratory for Biomedical Measurements and Ultrasound Imaging National‐Regional Key Technology Engineering Laboratory for Medical Ultrasound School of Biomedical Engineering Shenzhen University Medical School Shenzhen 518060 China; ^3^ Medical Research Center The Affiliated Yue Bei People's Hospital Shantou University Medical College Shaoguan 512025 China; ^4^ Basic Medical Science Department Zhuhai Campus of Zunyi Medical University Zhuhai 519041 China; ^5^ Oncology Department The Eighth Affiliated Hospital Sun Yat‐sen University Shenzhen 518060 China; ^6^ Institute of Otorhinolaryngology and Shenzhen Key of Otorhinolaryngology Longgang Otorhinolaryngology Hospital Shenzhen 518172 China; ^7^ The Bio‐bank of Shenzhen Second People's Hospital The First Affiliated Hospital of Shenzhen University Shenzhen Guangdong 518000 China; ^8^ Institute of Basic Medicine and Forensic Medicine North Sichuan Medical College Nanchong Sichuan 637199 China

**Keywords:** E‐cadherin, metastasis, nasopharyngeal carcinoma (NPC), phosphoglycerate kinase 1 (PGK1), TMEM52B isoforms

## Abstract

Transmembrane protein 52B (TMEM52B), a newly identified tumor‐related gene, has been reported to regulate various tumors, yet its role in nasopharyngeal carcinoma (NPC) remains unclear. Transcriptomic analysis of NPC cell lines reveals frequent overexpression of TMEM52B, and immunohistochemical results show that TMEM52B is associated with advanced tumor stage, recurrence, and decreased survival time. Depleting TMEM52B inhibits the proliferation, migration, invasion, and oncogenesis of NPC cells in vivo. TMEM52B encodes two isoforms, TMEM52B‐P18 and TMEM52B‐P20, differing in their N‐terminals. While both isoforms exhibit similar pro‐oncogenic roles and contribute to drug resistance in NPC, TMEM52B‐P20 differentially promotes metastasis. This functional discrepancy may be attributed to their distinct subcellular localization; TMEM52B‐P18 is confined to the cytoplasm, while TMEM52B‐P20 is found both at the cell membrane and in the cytoplasm. Mechanistically, cytoplasmic TMEM52B enhances AKT phosphorylation by interacting with phosphoglycerate kinase 1 (PGK1), fostering NPC growth and metastasis. Meanwhile, membrane‐localized TMEM52B‐P20 promotes E‐cadherin ubiquitination and degradation by facilitating its interaction with the E3 ubiquitin ligase NEDD4, further driving NPC metastasis. In conclusion, the TMEM52B‐P18 and TMEM52B‐P20 isoforms promote the metastasis of NPC cells through different mechanisms. Drugs targeting these TMEM52B isoforms may offer therapeutic benefits to cancer patients with varying degrees of metastasis.

## Graphical abstract

TMEM52B represents a promising biomarker for early diagnosis and prognostic prediction in nasopharyngeal carcinoma (NPC). Mechanistically, cytoplasmic TMEM52B‐P18 and TMEM52B‐P20 promote AKT phosphorylation by interacting with PGK1, thereby activating AKT signaling and driving NPC Carcinoma. On the other hand, membrane‐bound TMEM52B‐P20 induces the ubiquitination of E‐cadherin by NEDD4 and degrades it, thus promoting metastasis.

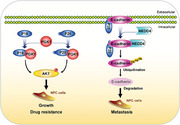

## 1 Introduction

Nasopharyngeal carcinoma (NPC) is a complex disease that involves different etiologic factors, including genetic susceptibility, Epstein‐Barr virus (EBV) infection, and environmental exposures.^[^ ^1^, ^2^, ^3^, ^4^, ^5^ ^]^

Conventional treatments for NPC include radiotherapy alone and concurrent chemoradiotherapy, but ≈20−30% of cases relapse, and re‐irradiation can be associated with significant debilitation. Additionally, these treatments have limited effects on local recurrence or distant metastasis.^[^ ^6^ ^]^ Despite the potential of immunotherapy as a salvage treatment for recurrent/metastatic NPC, its response rate is unsatisfactory.

In China, poorly differentiated and undifferentiated non‐keratinizing NPC are the main pathological subtypes, with Epstein‐Barr virus (EBV) infection being a unique etiological culprit agent.^[^ ^4^ ^]^ No licensed EBV prophylactic EBV vaccine is currently available yet licensed for NPC prevention. Studies have shown that EBV‐driven latent membrane protein 1 (LMP1)‐induced programmed death‐ligand 1 (PD‐L1), which is a promising therapeutic target.^[^ ^7^ ^]^ On the other hand, single‐cell transcriptomics studies to screen therapeutic targets for NPC have found that LAG3 and HAVCR2 may be more promising immunotherapeutic targets than PD1.^[^ ^8^ ^]^ Although these studies identified several targets for drug action, it is still contentious which may be the most advantageous and suitable for patients.

Given the current limitations of current therapeutic methods and the lack of effective drug targets, identifying novel oncogenic factors that contribute to NPC progression and metastasis and improving our understanding of the underlying molecular mechanisms, may help in developing more effective targeted therapies.^[^ ^9^, ^10^ ^]^

Transmembrane protein 52B (TMEM52B) (TMEM52B; gene ID, 120 939; chromosome location, 12p13.2; also termed C12orf59, FLJ31166 or MGC111385) was initially cloned in 2002 and is widely expressed in various normal human tissues, with its highest expression in the kidney.^[^ ^11^, ^12^ ^]^ Our previous findings revealed that decreased TMEM52B expression is associated with poor prognosis and VHL mutation in human renal cell carcinoma (RCC),^[^ ^12^ ^]^ indicating its role as a tumor suppressor in renal carcinogenesis. In a recent study, TMEM52B was found to function as a tumor suppressor in colon cancer, where overexpression of both TMEM52B isoform 1 and isoform 2 reduced tumor growth and early metastasis in an EGFR‐dependent manner.^[^ ^13^ ^]^ Despite current studies supporting a tumor‐suppressive role of TMEM52B in RCC and colon cancer, our recent sequencing results on NPC cells and normal nasopharyngeal cells show that TMEM52B is significantly upregulated in NPC cells, motivating us to investigate its potential oncogenic function in NPC. We also explored the differential functions between TMEM52B isoforms and found that TMEM52B‐P18 favored NPC growth, while TMEM52B‐P20 tended to motivate NPC metastasis. Our findings suggested that TMEM52B could serve as a promising biomarker and therapeutic target in NPC.

## 2 Results

### 2.1 TMEM52B is Upregulated in NPC and Associated with Poor Prognosis

**Figure 1:**
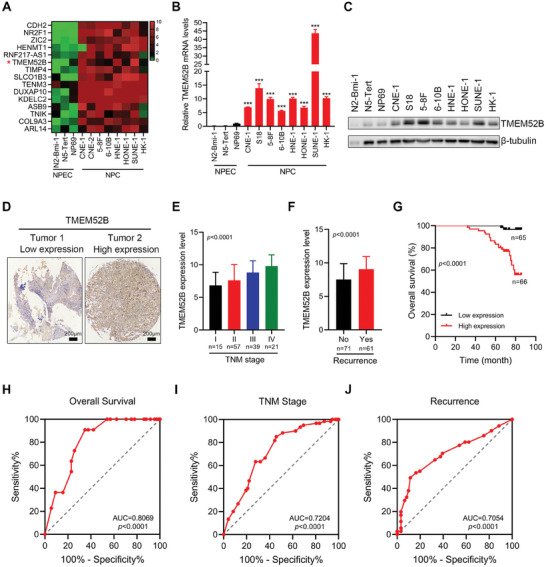
Identification of TMEM52B, upregulation of which in NPC is correlated with poor prognosis. A) Heatmap of the top 15 differentially expressed genes from RNA sequencing that were upregulated in various types of NPC cells. B) mRNA levels of TMEM52B by real‐time PCR in NPEC and NPC cells with β‐actin as the internal control. Data were represented as mean ± standard deviation (SD), n = 3, ^***^ *p* < 0.001 versus NP‐69. C) Protein levels of TMEM52B by western blotting in NPEC and NPC cells, β‐tubulin was used as the internal control. D) Immunohistochemical staining of TMEM52B expression in NPC using a tissue microarray containing 132 human NPC tissues. Scale bar, 200 µm. E,F) TMEM52B expression was analyzed in NPC patients regarding tumor node metastasis (TNM) stage and recurrence using data in the NPC tissue microarray. G) Overall survival in NPC patients based on the expression of TMEM42B in the NPC tissue microarray, shown by Kaplan–Meier analysis. The median of TMEM52B levels was used to divide the high and low‐expression groups. H) TMEM52B was a biomarker for the overall survival of NPC as indicated by receiver operating curve (ROC) analysis. I,J) ROC curve analysis for TMEM52B as a diagnostic indicator in discriminating advanced stages (stages III and IV) from early stages (stages I and II), and to predict recurrence from no recurrence for NPC patients. *P* value calculated by unpaired two‐tailed Student's *t*‐test (B, F), one‐way ANOVA (E), and log‐rank test(G).

To explore the role of tumor‐related gene TMEM52B in NPC, we conducted transcriptome sequencing of three Normal nasopharyngeal epithelial cell (NPEC) and eight NPC cell lines. We identified differentially expressed genes. Among the 3415 upregulated genes, TMEM52B, previously identified in our report as a tumor suppressor in RCC,^[^ ^12^ ^]^ showed a significant increase of more than 20‐fold (2^4.66^) in all eight NPC cell lines compared to normal NPEC cells (**Figure** **1**A) (Data S1, Supporting Information), real‐time PCR (Figure 1B) and western blotting (Figure 1C) confirmed this observation.

We next performed immunohistochemistry on a tissue microarray containing 132 human NPC tissues to determine the clinical relevance of TMEM52B expression in NPC. Based on the expression levels of TMEM52B in cancer tissues, we divided all NPC patients into high and low expression groups (Figure 1D); high TMEM52B expression was significantly associated with advanced tumor node metastasis stage and recurrence (Figure 1E,F). Furthermore, Kaplan‐Meier survival analysis showed that high TMEM52B expression correlated negatively with survival in NPC patients (Figure 1G), and Cox regression analysis indicated that TMEM52B expression was an independent risk factor for survival (**Table** **1**). Receiver operating curve analysis also demonstrated that TMEM52B expression was a predictive marker for overall survival (Figure 1H) and recurrence versus no recurrence in NPC patients (Figure 1J), and was a diagnostic indicator for discriminating advanced stages (stages III and IV) from early stages (stages I and II) (Figure 1I). These findings suggest that high TMEM52B expression in NPC is associated with poor clinical outcomes.

**Table 1:** Univariate and multivariate Cox regression analyses of TMEM52B for overall survival in NPC.

Variable	Univariate analysis			Multivariate analysis		
	HR	95% *CI*	P value	HR	95% *CI*	P value
TMEM52B expression (high vs low)	12.981	3.021–55.783	<0.001	8.840	2.029‐38.511	0.004
TNM stage (T3‐T4 vsT1‐T2)	13.561	3.169–58.040	<0.001	9.255	2.146‐39.911	0.003
Lymph node metastasis (yes vs no)	8.404	1.130–62.488	0.038			
Age (≥60 vs <60 years)	0.743	0.220–2.511	0.633			
Gender (Female vs male)	0.952	0.351–2.583	0.924			

### 2.2 TMEM52B Depletion Inhibits NPC Cell Proliferation, Migration, and Invasion

**Figure 2:**
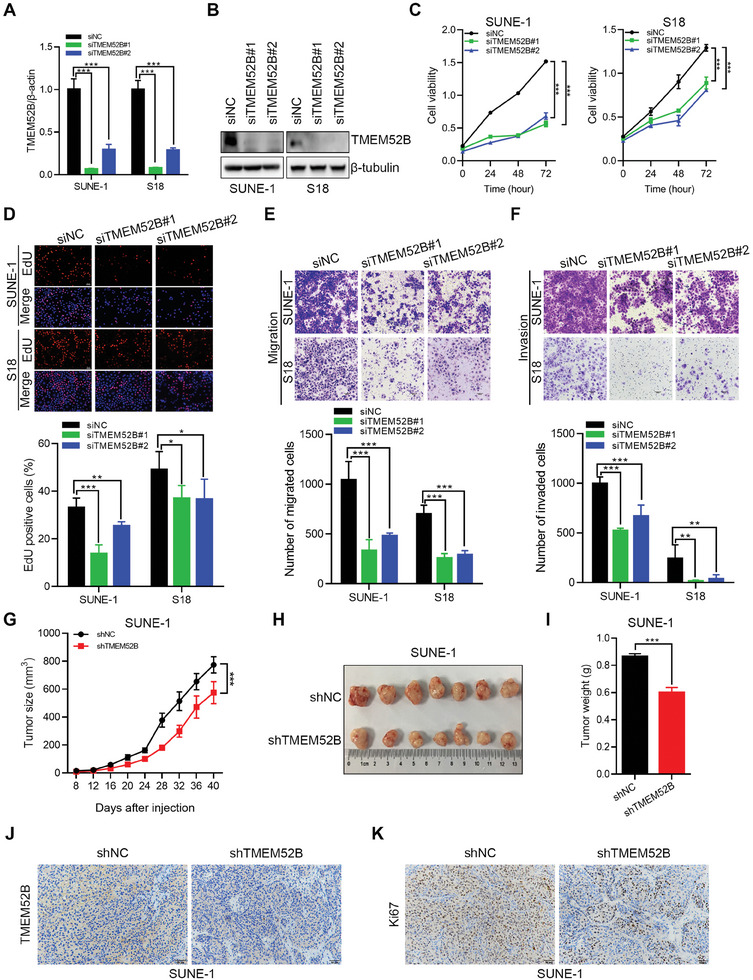
TMEM52B knockdown inhibits NPC growth and metastasis in vitro and in vivo. A,B) Decreased mRNA and protein levels of TMEM52B in SUNE‐1 and S18 cells following siRNA transfection were shown by real‐time PCR and western blotting, β‐actin and β‐tubulin were used as the internal control, respectively C,D) The inhibitory effects on cell viability and proliferation after TMEM52B knockdown as shown by CCK‐8 and EdU assays in SUNE‐1 and S18 cells. Scale bar, 50 µm. E,F) Transwell assays to determine the suppressive effects of TMEM52B depletion on the migration and invasion of SUNE‐1 and S18 cells. Scale bar, 100 µm. G–K) SUEN‐1 cells pre‐infected with control or TMEM52B‐shRNA‐expressing lentivirus were subcutaneously inoculated into the flank of each BALB/c nude mouse (*n* = 7 per group). The growth curves of tumors (G), tumor photographs (H); and tumor weights (I), in the nude mice were analyzed. The expression of TMEM52B (J) and Ki67 (K) in subcutaneous tumors was estimated using immunohistochemistry. Scale bar, 50 µm. Data were represented as mean ± SD in (A,C–F), n≥3, and mean ± standard error of the mean (SEM) in (G,I), *p < 0.05, **p < 0.01, ***p < 0.001. *P* value calculated by unpaired two‐tailed Student's *t* test (A,D–F,I), and two‐way ANOVA (C, G).

We next generated SUNE‐1 shTMEM52B and S18 shTMEM52B cell lines, which were infected with TMEM52B‐shRNA‐expressing lentiviral vector (LV‐shTMEM52B) and expressing TMEM52B at a stable low level, to investigate the potential role of TMEM52B in NPC. We first confirmed that both TMEM52B mRNA and protein levels were significantly decreased in SUNE‐1 and S18 cells following TMEM52B knockdown (**Figure** **2**A,B). TMEM52B depletion markedly inhibited cell viability (CCK‐8 assay; Figure 2C), NPC cell proliferation (EdU assay; Figure 2D), and NPC cell migration and invasion (transwell assay; Figure 2E,F).

To investigate whether TMEM52B influences NPC tumor growth in vivo, we performed xenograft experiments in BALB/c nude mice via subcutaneous inoculation of SUNE‐1 cells transfected with either control‐shRNA‐expressing lentiviral vector (LV‐shCtrl) or LV‐shTMEM52B. As expected from our in vitro analyses, the volume and weight of the LV‐shTMEM52B tumors were significantly reduced compared with those of the LV‐shCtrl xenografts (Figure 2G–I). Furthermore, immunohistochemistry showed notably decreased TMEM52B and Ki67 expression in LV‐shTMEM52B xenograft tumors (Figure 2J,K). Together, these results demonstrate that TMEM52B knockdown significantly inhibits NPC tumor growth and metastasis.

### 2.3 TMEM52B Isoforms Differentially Promote Tumor Growth and Metastasis

**Figure 3:**
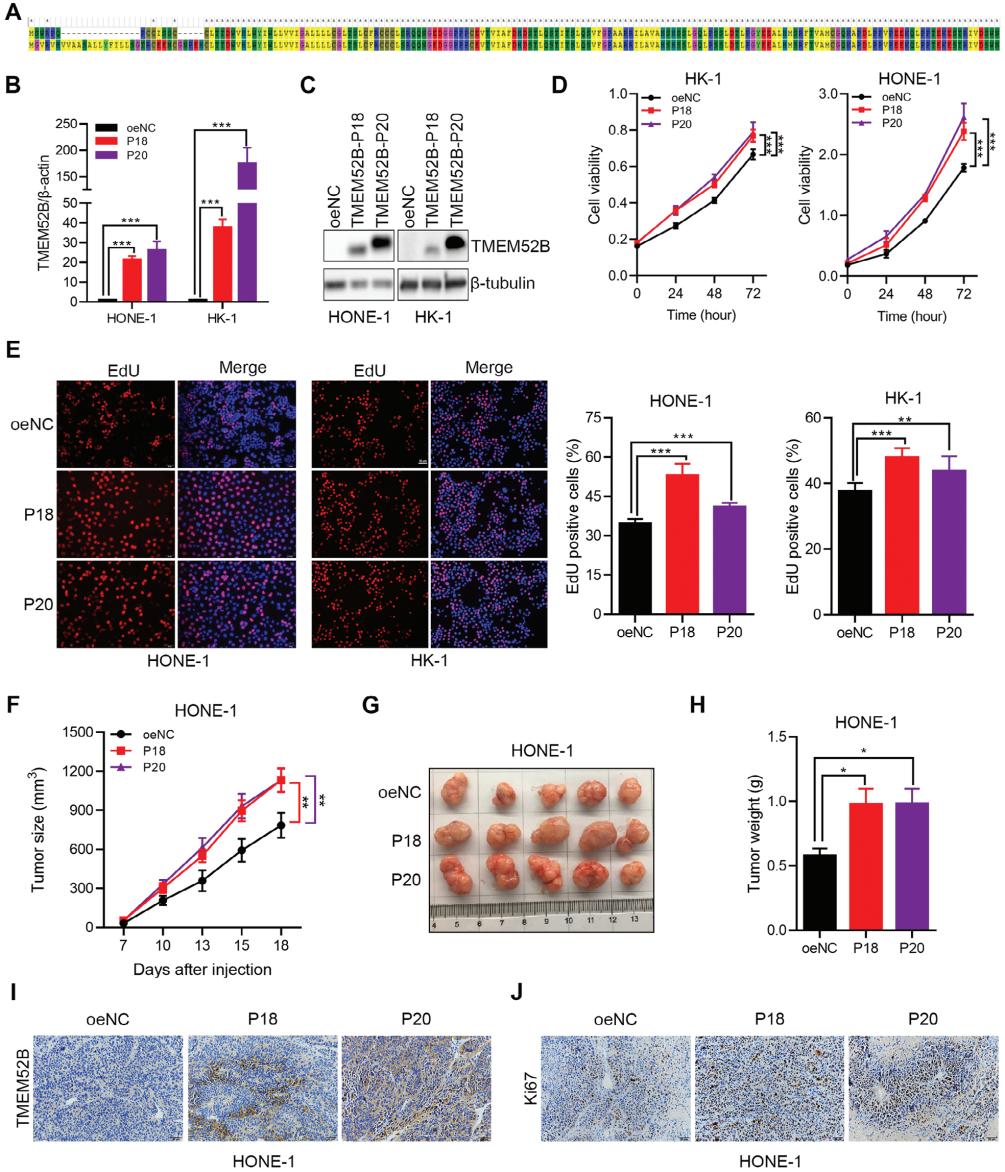
Overexpression of TMEM52B‐P18 and TMEM52B‐P20 isoforms similarly promotes NPC growth in vitro and in vivo. A) The aligned amino acid sequences of TMEM52‐B‐P18 and TMEM52B‐P20 isoforms. B,C) The increased mRNA and protein levels of TMEM52B isoforms in HONE‐1 and HK‐1 cells following infection with control, TMEM52B‐P18‐expressing, or TMEM52B‐P20‐expressing lentivirus, analyzed by real‐time PCR and western blotting with β‐actin and β‐tubulin as the internal control, respectively. D,E) The role of TMEM52B isoforms in promoting cell viability and proliferation after TMEM52B overexpression were shown using CCK‐8 and EdU assays in HONE‐1 and HK‐1 cells. Scale bar, 50 µm. F–J. HONE‐1 cells pre‐infected with control, TMEM52B‐P18‐expressing, or TMEM52B‐P20‐expressing lentivirus were subcutaneously inoculated into the flank of each BALB/c nude mouse (*n* = 5 per group). The growth curves of F) tumors, G) tumor photographs, and H) tumor weights are shown. The expression of I) TMEM52B and J) Ki67 in subcutaneous tumors was estimated using immunohistochemistry. Scale bar, 50 µm. Data were represented as mean ± SD in (B, D, E), n≥3, and mean ± SEM in (F,H), *p < 0.05, **p < 0.01, ***p < 0.001. P value calculated by unpaired two‐tailed Student's *t*‐test (B,E,H), and two‐way ANOVA (D,F).

There are two known TMEM52B isoforms.^[^ ^13^ ^]^ TMEM52B isoform 1 encodes an 18 kDa, 163 amino acid protein, while isoform 2 encodes a 20 kDa, 183 amino acid protein with a distinct N‐terminal (**Figure** **3**A). To distinguish the roles of these isoforms in NPC, we generated stable cell lines overexpressing TMEM52B‐P18 and TMEM52B‐P20 (Figure 3B,C). Both TMEM52B‐P18 and TMEM52B‐P20 significantly promoted the proliferation of HONE‐1 and HK‐1 cells in vitro, as shown by CCK‐8 and EdU assays (Figure 3D,E). In vivo, both LV‐TMEM52B‐P18 and LV‐TMEM52B‐P20 tumors in BALB/c nude mice were larger and heavier than LV‐Ctrl tumors, with significantly more Ki‐67 positive cells (Figure 3F–J). These data suggest that both of the TMEM52B isoforms promote NPC tumor growth.

**Figure 4:**
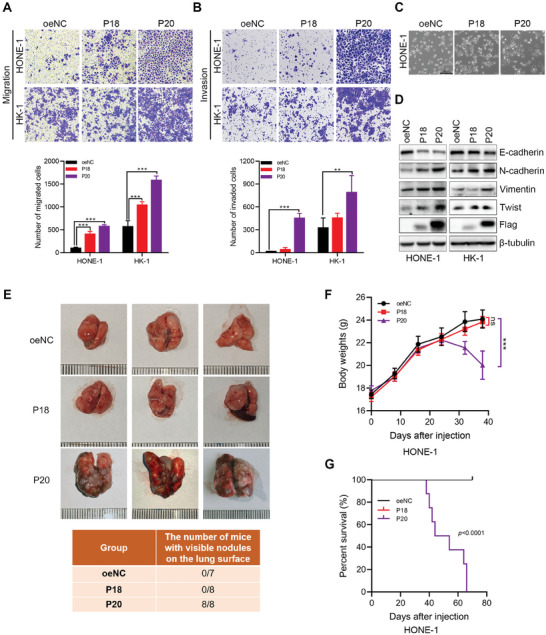
Overexpressing TMEM52B‐P18 and TMEM52B‐P20 isoforms differentially promotes NPC metastasis in vitro and in vivo. A,B) Transwell assays to determine the effects of TMEM52B isoforms after TMEM52B overexpression on accelerating the migration and invasion of HONE‐1 and HK‐1 cells. Scale bar, 100 µm. C) Representative images showed different cell morphology of HONE‐1 cells overexpressing TMEM52B isoforms versus controls. Scale bar, 200 µm. D) Protein levels of E‐cadherin, N‐cadherin, vimentin, and twist in cells overexpressing TMEM52B‐P18, or TMEM52B‐P20 and in controls were analyzed by western blotting using whole lysates extracted from HONE‐1 and HK‐1 cells, β‐tubulin was used as the loading control. E) Photographs of pulmonary tumors generated by HONE‐1 cells transfected with LV‐oeCtrl (*n* = 7), LV‐TMEM52B‐P18 (*n* = 8) or LV‐TMEM52B‐P20 (*n* = 8). F) Body weight curves of nude mice injected with LV‐oeCtrl, LV‐TMEM52B‐P18, or LV‐TMEM52B‐P20 HONE‐1 cells. G. Survival curves of nude mice inoculated with LV‐oeCtrl, LV‐TMEM52B‐P18, or LV‐TMEM52B‐P20 HONE‐1 cells. Data were represented as mean ± SD, n = 5, in (A,B) and mean ± SEM in (F); ns, no significance; **p < 0.01, ***p < 0.001. P value calculated by unpaired two‐tailed Student's *t*‐test (A,B), two‐way F) ANOVA, and G) log‐rank test.

We also saw that overexpression of both TMEM52B‐P18 and TMEM52B‐P20 significantly enhanced the migratory and invasive capacities of HONE‐1 and HK‐1 cells in transwell assays, with TMEM52B‐P20 having a significantly stronger effect (**Figure** **4**A,B). HONE‐1 cells overexpressing TMEM52B‐P20 also had a distinct spindle‐like morphology compared to TMEM52B‐P18 overexpressing or control cells (Figure 4C). This suggests that overexpression of TMEM52B‐P20 may affect the function of cytoskeletal protein. In view of this, we also found the interaction between TMEM52B and PLEC‐1 in preliminary experiments (Figure S1A, Supporting Information). Furthermore, overexpression of both TMEM52B‐P18 and TMEM52B‐P20 induced epithelial‐mesenchymal transition (EMT) in HONE‐1 cells, with higher protein levels of N‐cadherin, vimentin, and twist, and lower protein levels of E‐cadherin compared to control cells (Figure 4D).

We finally established an in vivo pulmonary metastasis tumor model using male BALB/c nude mice, and observed that the LV‐TMEM52B‐P20 group formed significantly more tumor nodules in lung tissues compared with the LV‐TMEM52B‐P18 group, and that mice with LV‐TMEM52B‐P20 tumors showed a rapid loss of body weight and decreased survival time (Figure 4E–G). Neither LV‐Ctrl group nor LV‐TMEM52B‐P18 group died, so the survival curves overlapped. These findings support that TMEM52B‐P20 likely has a stronger effect than TMEM52B‐18 in promoting NPC metastasis.

### 2.4 TMEM52B Isoforms Stimulate NPC Development by Affecting AKT Phosphorylation

**Figure 5:**
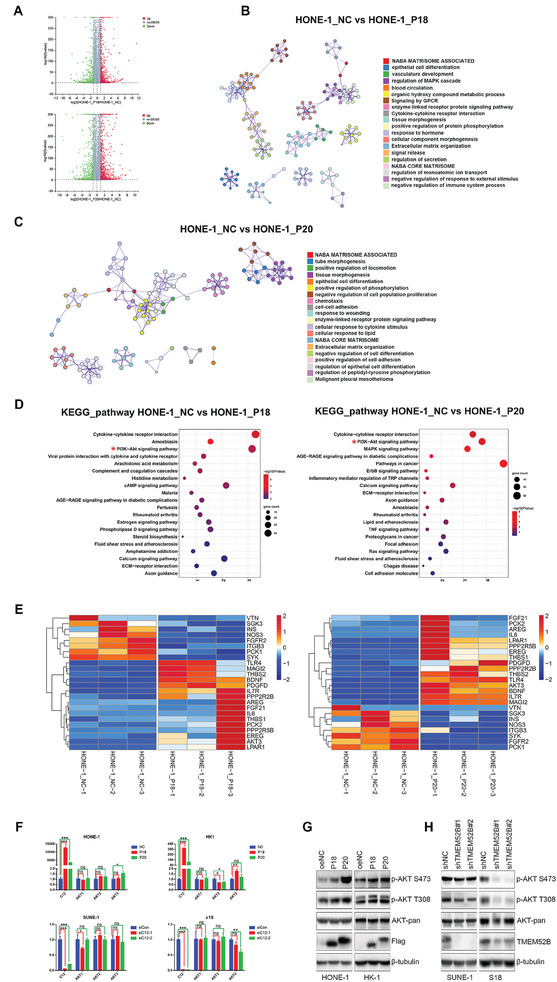
RNA sequencing reveals that TMEM52B affects AKT signaling. A) Volcano plots of RNA sequencing analysis of HONE‐1 cells pre‐infected with control, TMEM52B‐P18‐expressing, or TMEM52B‐P20‐expressing lentivirus (|log2fold change| > 1 and Q value < 0.01, green indicates downregulated genes; red indicates upregulated genes). B,C) Metascape enrichment analysis showing groups of several categories based on gene functional relevance; a network was constructed based on relevance and similarity. Different colors represent different categories. D) The top 20 enriched biological processes of differentially expressed genes in LV‐TMEM52B‐P18/LV‐oeCtrl and LV‐TMEM52B‐P20/LV‐oeCtrl HONE‐1 cells. E) Heat map showing the expression of PI3K‐Akt signaling pathway‐related genes by HONE‐1 cells pre‐infected with control, TMEM52B‐P18‐expressing, or TMEM52B‐P20‐expressing lentivirus (blue indicates downregulated genes; red indicates upregulated genes). F) Real‐time PCR analysis of mRNA levels of AKT and TMEM52B in HONE‐1 or HK‐1 cells overexpressing TMEM52B isoforms and SUNE‐1 or S18 cells silencing TMEM52B, β‐actin was used as the internal control. G,H) Phosphorylated levels of AKT at S473 and T308 sites in LV‐oeCtrl, LV‐TMEM52B‐P18 or LV‐TMEM52B‐P20 HONE‐1 and HK‐1 cells (G), as well as LV‐shCtrl or LV‐shTMEM52B infected SUNE‐1 and S18 cells (H), by western blotting with β‐tubulin as the loading control. Data were represented as mean ±SD, n≥3; ns, no significance; *p < 0.05, **p < 0.01, ***p < 0.001. P value calculated by unpaired two‐tailed Student's *t* test.

To investigate how the two TMEM52B isoforms promote cell proliferation, migration, and invasion in NPC, we performed transcriptome sequencing on HONE‐1 cells overexpressing each of the TMEM52B isoforms and on control cells. We identified 1499 differentially expressed genes, including 661 downregulated and 838 upregulated genes, in HONE‐1 cells overexpressing TMEM52B‐P18, and 1704 differentially expressed genes, including 725 downregulated and 979 upregulated genes, in HONE‐1 cells overexpressing TMEM52B‐P20 (**Figure** **5**A), an additional table file shows this in more detail (Data S2, Supporting Information). Metascape enrichment analysis showed that these genes encode proteins that regulate a variety of important cellular functions and form complicated regulatory networks. We focused on those associated with cell proliferation and metastasis, which are critical in oncogenesis and tumor development (Figure 5B,C). Gene ontology enrichment analyses revealed that the AKT signaling pathway was one of the most altered pathways in HONE‐1 cells overexpressing TMEM52B‐P18 and TMEM52B‐P20 (Figure 5D). Heatmap analysis further confirmed that the most differentially expressed genes were involved in the PI3K‐Akt signaling pathway (Figure 5E).

To analyze the PI3K‐Akt signaling pathway in more detail, we performed a real‐time PCR analysis to measure AKT mRNA in HONE‐1 cells overexpressing either TMEM52B‐P18 or TMEM52B‐P20, as well as in control cells. We saw no significant changes in AKT mRNA levels between the groups (Figure 5F), but the TMEM52B protein levels did have an impact on AKT phosphorylation. Specifically, overexpression of TMEM52B‐P18 or TMEM52B‐P20 significantly induced AKT phosphorylation at S473 and T308 sites in HONE‐1 and HK‐1 cells (Figure 5G), while TMEM52B knockdown in SUNE‐1 and S18 cells led to an apparent inhibition of AKT phosphorylation (Figure 5H). Meanwhile changes in AKT downstream proteins mTOR, p21 and p27 further confirm that TMEM52B has a role in regulating the AKT signaling pathway (Figure S1B, Supporting Information).

### 2.5 TMEM52B Promotes NPC Proliferation by Enhancing PGK1‐Mediated AKT Phosphorylation

**Figure 6:**
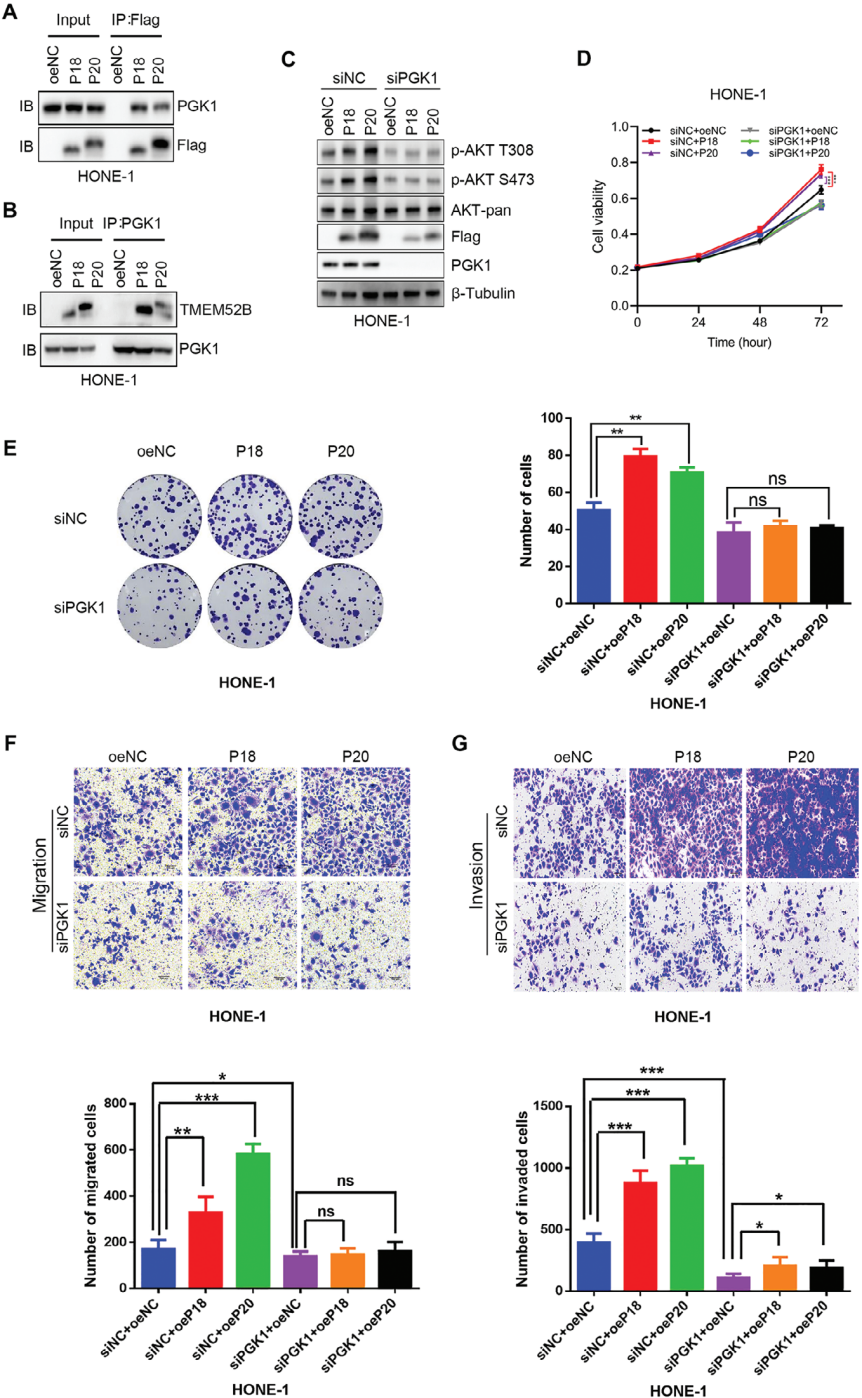
TMEM52B isoforms interact with PGK1 to promote NPC growth. A) Both TMEM52B isoforms interact with PGK1. Cell lysates from HONE‐1 cells overexpressing TMEM52B‐P18 or TMEM52B‐P20 and control cells were immunoprecipitated with anti‐Flag, followed by western blotting with anti‐PGK1 or anti‐Flag antibody on the precipitates and lysates, as indicated. B) Reciprocal immunoprecipitation (IP) was performed on cell lysates from HONE‐1 cells overexpressing TMEM52B‐P18 or TMEM52B‐P20 and control cells, by precipitating with anti‐PGK1, followed by western blotting with anti‐TMEM52B or anti‐PGK1 antibody on the precipitates and lysates, as indicated. C) The effects of TMEM52B‐P18 and TMEM52B‐P20 on the phosphorylation of AKT depend on PGK1. HONE‐1 cells were transfected with siRNA control or PGK1 siRNA sequences, followed by the transfection of control, TMEM52B‐P18 or TMEM52B‐P20 plasmids. Western blotting was performed to estimate the phosphorylation of AKT at S473 and T308 sites with β‐tubulin as the loading control. D) Cell viability and proliferation of HONE‐1 cells with PGK1 silencing followed by TMEM52B‐P18 or TMEM52B‐P20 overexpression, assessed by CCK‐8. E) Cloning of HONE‐1 cells with PGK1 silencing followed by TMEM52B‐P18 or TMEM52B‐P20 overexpression. F,G) Cell migration and invasion of HONE‐1 cells with PGK1 silencing followed by TMEM52B‐P18 or TMEM52B‐P20 overexpression, assessed by transwell assays. Scale bar, 100 µm. Data were represented as mean ±SD, n ≥ 3; ns, no significance; *p < 0.05, **p < 0.01, ***p < 0.001. P value calculated by unpaired two‐tailed Student's t test (E‐G) and two‐way ANOVA (D).

Having shown the impact of TMEM52B on AKT phosphorylation, we next decided to investigate the intrinsic mechanism by which TMEM52B regulates AKT phosphorylation. To do so, we conducted co‐immunoprecipitation and mass spectrometry experiments to identify TMEM52B‐interacting proteins in HONE‐1 cells. Coomassie Brilliant Blue protein assay revealed specific binding proteins in HONE‐1 cells overexpressing TMEM52B‐P18 and TMEM52B‐P20 (Figure S1C, Supporting Information). Mass spectrometry analysis subsequently showed that phosphoglycerate kinase 1 (PGK1) co‐precipitated with both TMEM52B‐P18 and TMEM52B‐P20 in HONE‐1 cells (Data S3, Supporting Information). Further investigation of TMEM52B isoform interactions with PGK1 revealed that the two proteins indeed bind to each other (**Figure** **6**A,B).

PGK1 is an ATP‐generating enzyme that activates the AKT/mTOR pathway in non‐small cell lung cancer.^[^ ^14^ ^]^ To determine whether PGK1 is involved in TMEM52B‐dependent AKT phosphorylation and oncogenic behaviors such as proliferation, migration, and invasion, we designed siRNA oligonucleotides targeting PGK1 and introduced them into HONE‐1, then overexpressed both of the TMEM52B isoforms. PGK1 ablation significantly suppressed increases in phosphorylated AKT S473 and T308 levels induced by TMEM52B‐P18 and TMEM52B‐P20 (Figure 6C). Moreover, PGK1 knockdown impaired the enhanced proliferative capability of HONE‐1 cells overexpressing TMEM52B‐P18 and TMEM52B‐P20, as shown by CCK‐8 (Figure 6D) and colony formation assays (Figure 6E). Transwell assays further demonstrated that PGK1 knockdown significantly suppressed the increased migratory and invasive capabilities of HONE‐1 cells due to TMEM52B overexpression (Figure 6F,G). We thus propose that interactions between PGK1 and TMEM52B isoforms are probably involved in activating AKT signaling and downstream cell proliferation, migration, and invasion in NPC.

### 2.6 TMEM52B Isoform Localization Differentially Affects NPC Cell Migration and Invasion

**Figure 7:**
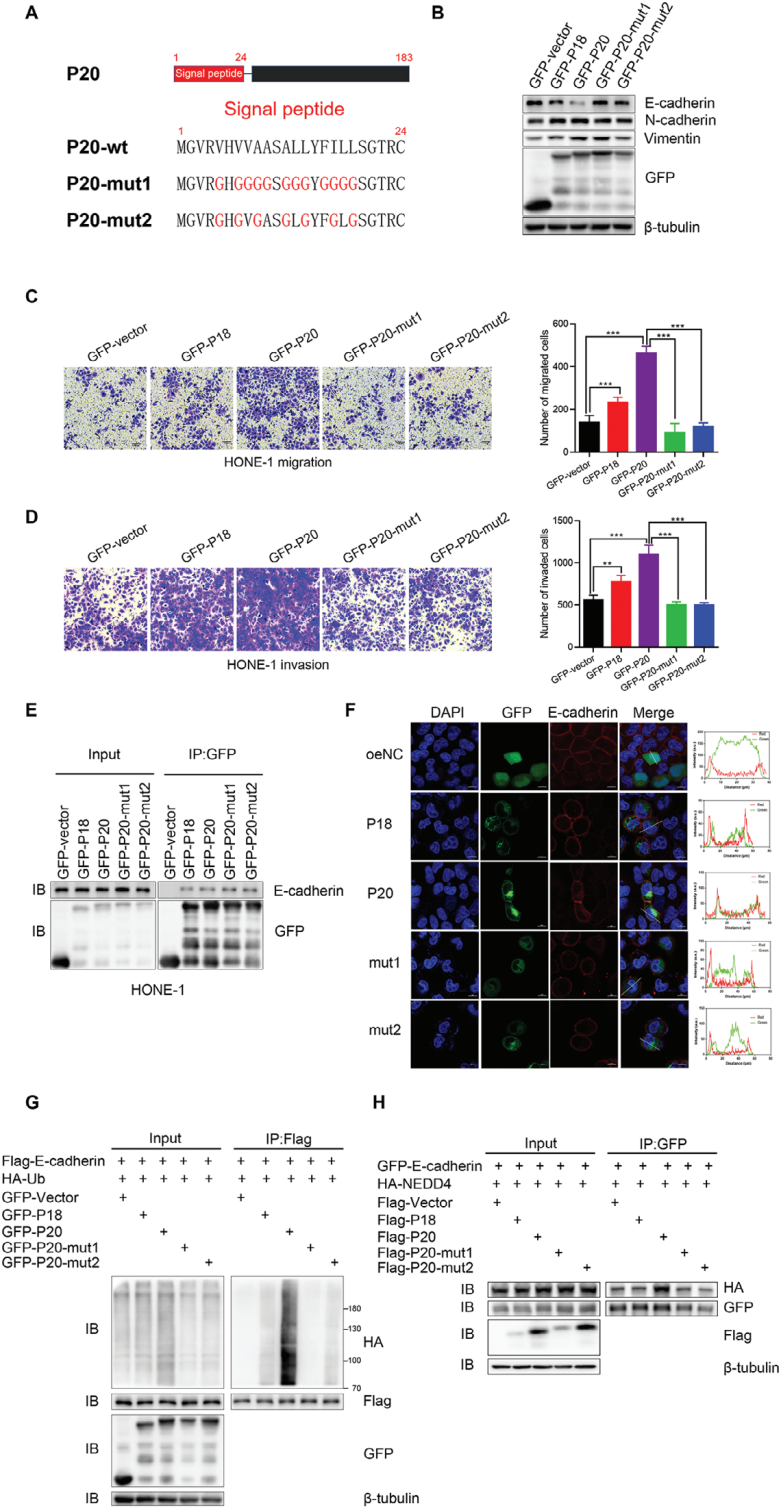
Plasma membrane‐associated TMEM52B‐P20 markedly promotes NPC metastasis. A) Schematic diagram of P20 mutants. B) Western blotting to determine the influence of TMEM52B‐P20 mutants on the protein levels of E‐cadherin, N‐cadherin, and vimentin in HONE‐1 cells. C,D) The effects of TMEM52B‐P20 mutants that dissociated TMEM52B‐P20 from the plasma membrane on the migratory (C) and invasive (D) capabilities of HONE‐1 cells, assessed by transwell assays. Scale bar, 100 µm. E) TMEM52B interacts with cell membrane protein. Cell lysates from HONE‐1 cells transfected with control, TMEM52B‐P18, TMEM52B‐P20, or TMEM52B‐P20 mutant plasmids were immunoprecipitated with anti‐GFP to pull down TMEM52B‐P18 and TMEM52B‐P20, followed by western blotting with anti‐E‐cadherin or anti‐GFP antibody on the precipitates and lysates, as indicated. F) Co‐localization of E‐cadherin with GFP‐P20 but not GFP‐P18 or GFP‐P20 mutants by immunostaining analysis. Scale bar, 10 µm. G) TMEM52B‐P20 increases the ubiquitination level of E‐cadherin. Cell lysates from HONE‐1 cells transfected with Flag‐E‐cadherin, HA‐Ub, and TMEM52B plasmids were immunoprecipitated with anti‐Flag to pull down ubiquitinated E‐cadherin, followed by western blotting with anti‐Flag, anti‐GFP or anti‐HA antibody on the precipitates and lysates, as indicated. H) TMEM52B‐P20 promotes the interaction of E‐cadherin with NEDD4. Cell lysates from HONE‐1 cells transfected with GFP‐E‐cadherin, HA‐NEDD4, and TMEM52B plasmids were immunoprecipitated with anti‐GFP to pull down HA‐NEDD4, followed by western blotting with anti‐Flag, anti‐GFP or anti‐HA antibody on the precipitates and lysates, as indicated. Data were represented as mean ±SD, n = 5, **p <0.01, ***p < 0.001. P value calculated by unpaired two‐tailed Student's *t* test.

Our in vitro and in vivo experiments thus far indicated that TMEM52B‐P20 promotes NPC cell motility and metastasis more effectively than TMEM52B‐P18, prompting us to investigate the underlying mechanisms. Although both TMEM52B isoforms share the same 150 amino acid C‐terminal sequence, the 33 amino acid N‐terminal sequence of TMEM52B‐P20 contains signal peptides (**Figure** **7**A), indicating that TMEM52B‐P20 is probably associated with the plasma membrane, while TMEM52B‐P18 is primarily cytoplasmic. Indeed, previous studies have shown that TMEM52B‐P20 is primarily detected at the cell membrane edges, while TMEM52B‐P18 is mainly found in the cytoplasm.^[^ ^13^ ^]^ To explore whether the different subcellular localizations of the TMEM52B isoforms accounts for their distinct roles in NPC, we generated GFP‐fused TMEM52B‐P20‐expression plasmids with mutations in the N‐terminal region; specifically, we introduced mutations in the hydrophobic amino acids of TMEM52B‐P20 to glycine in TMEM52B‐P20‐mut1, as well as partial mutations in TMEM52B‐P20‐mut2. Analysis of the GFP fluorescence showed that TMEM52B‐P18 was localized in the cytoplasm, while TMEM52B‐P20 was detected both in the cytosol and on the plasma membrane. However, when TMEM52B‐P20‐mut1 and TMEM52B‐P20‐mut2 were transfected into HONE‐1 cells, the plasma membrane localization of TMEM52B‐P20 was disrupted and it was only localized in the cytoplasm, (Figure S2A, Supporting Information). Western blotting results indicated that decreased E‐cadherin protein expression and increased levels of N‐cadherin and vimentin caused by wild‐type TMEM52B‐P20 overexpression, were partially restored by the transfected TMEM52B‐P20 mutants (Figure 7B). These findings suggest that the stronger effects of TMEM52B‐P20 in regulating cell function compared with TMEM52B‐P18 may depend on its differential plasma membrane localization, which is enabled by its unique N‐terminal region.

To investigate the role of plasma membrane‐associated TMEM52B‐P20 in metastasis, we conducted transwell assays on HONE‐1 cells transfected with wild‐type TMEM52B isoforms and TMEM52B‐P20 mutants. Overexpression of wild‐type TMEM52B‐P20 significantly enhanced the migratory and invasive abilities of HONE‐1 cells, whereas TMEM52B‐P20 mutants inhibited these effects (Figure 7C,D). Furthermore, although TMEM52B‐P18, TMEM52B‐P20, and the two mutants all have co‐localization with E‐cadherin (Figure 7E), the co‐localization of TMEM52B‐P20 and E‐cadherin on the cell membrane was markedly stronger than that of TMEM52B‐P18 and the two mutants (Figure 7F). Our studies further revealed that overexpression of TMEM52B‐P20 resulted in a significant reduction of E‐cadherin protein expression, but not transcription (Figure S2B, Supporting Information), and this suggested that the change of E‐cadherin expression level was caused by posttranslational modification of protein and was most likely ubiquitination modification. Subsequent experiments revealed that overexpression of TMEM52B‐P20 upregulated the ubiquitination of E‐cadherin, which explains the change in E‐cadherin expression (Figure 7G). We then overexpressed several reported E‐cadherin‐associated E3 ubiquitin ligases, NEDD4, MDM2, ring finger protein 43 (RNF43), and Cbl proto‐oncogene like 1 (Hakai) in HONE‐1 cells.^[^ ^15^, ^16^, ^17^, ^18^ ^]^We found that NEDD4 and MDM2 can reduce the stability of E‐cadherin (Figure S2C,D, Supporting Information), while RNF43 and Hakai do not have this effect in HONE‐1 cells (Figure S2E,F, Supporting Information). In further studies, we found that TMEM52B‐P20 can significantly improve the interaction between E‐cadherin and E3 ubiquitin ligase NEDD4 (Figure 7H), and slightly improve the interaction between E‐cadherin and E3 ubiquitin ligase MDM2 (Figure S2G, Supporting Information). The binding level of E‐cadherin with E3 ubiquitin ligase Hakai and RNF43 was almost not affected (Figure S2H,I, Supporting Information). These findings suggest that plasma membrane‐associated TMEM52B‐P20 has a critical role in NPC metastasis, by interacting with cell membrane protein E‐cadherin and regulating EMT signaling.

### 2.7 TMEM52B Enhances Drug Resistance in NPC Cells

**Figure 8:**
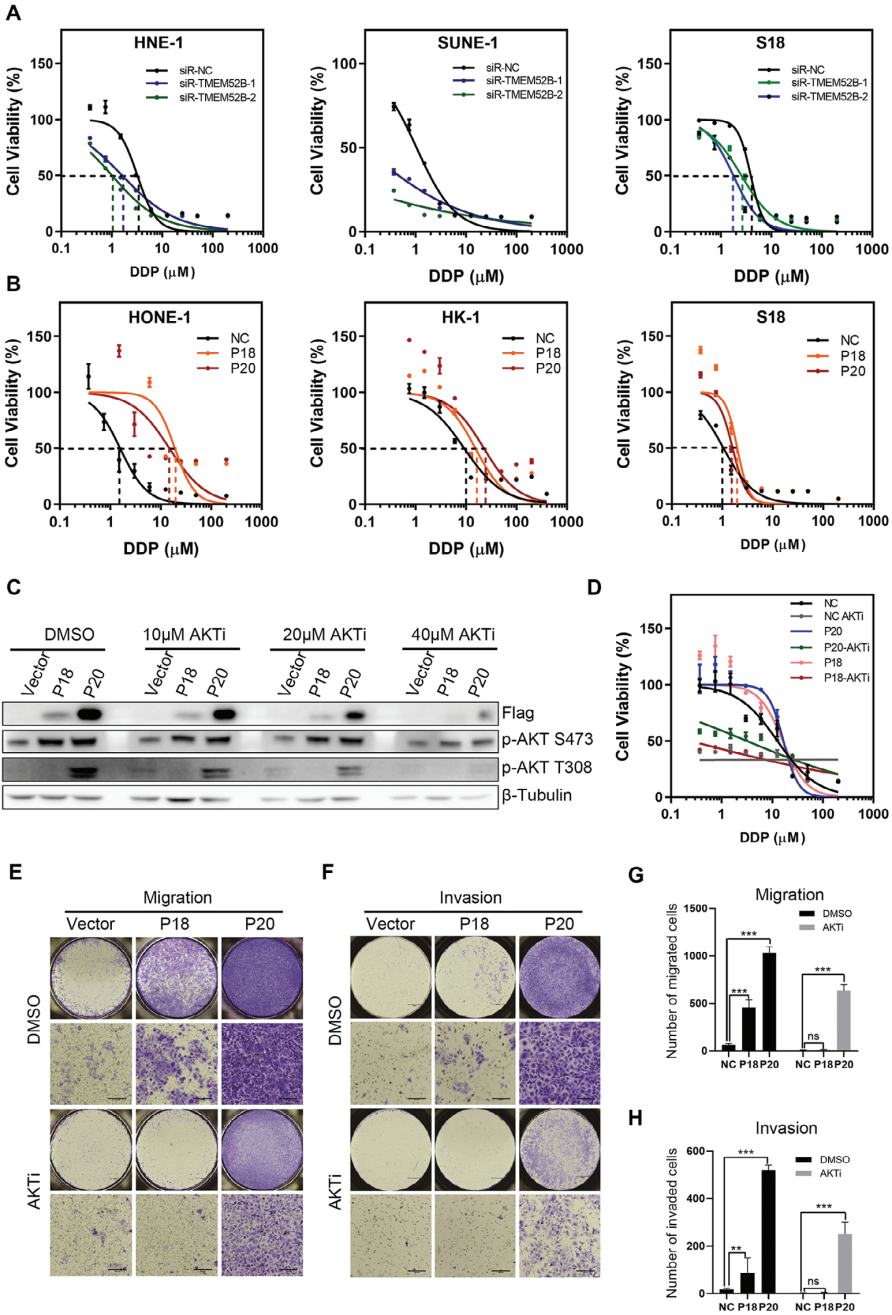
TMEM52B enhances drug resistance in NPC cells. A) HNE‐1, SUNE‐1 and S18 cells were transfected with small interfering RNA, and after 48 hours the cells were passed and treated with different concentrations of DDP. Cell survival rate was calculated and cell survival curve was drawn. B) HONE‐1, HK‐1 and S18 cells were transfected with TMEM52B‐P18 or TMEM52B‐P20, and after 48 hours the cells were passed and treated with different concentrations of DDP. Cell survival rate was calculated and cell survival curve was drawn. C) HONE‐1 cells were transfected with TMEM52B‐P18 or TMEM52B‐P20, and treated with different concentrations of AKT phosphorylation inhibitor. Cell lysates were detected by western blotting with anti‐Flag, anti‐pAKT S473 or anti‐pAKT T308. D) HONE‐1 cells were transfected with TMEM52B‐P18 or TMEM52B‐P20. 48 hours later the cells were passed and treated with different concentrations of DDP and 20 µm AKTi. Cell survival rate was calculated and cell survival curve was drawn. E‐H. Cell migration and invasion of HONE‐1 cells transfected with TMEM52B‐P18 or TMEM52B‐P20 followed by AKTi treatment, assessed by transwell assays. Scale bar, 100 µm. Data were represented as mean ±SD, n = 6; ns, no significance; **p < 0.01, ***p < 0.001. P value calculated by unpaired two‐tailed Student's *t* test.

Tumor drug resistance is another important cause of recurrence and death in NPC patients.^[^ ^19^ ^]^ Therefore, our work preliminarily investigated the role of TMEM52B in chemotherapy resistance of NPC cells. Here we treated the cells with cisplatin (DDP), a chemotherapy drug commonly used in the treatment of NPC.^[^ ^20^ ^]^ The results showed that after treating the cells with different concentrations of DDP, the chemotherapy drug sensitivity of NPC cells SUNE‐1, HNE‐1 and S18 with TMEM52B knockdown was significantly enhanced (**Figure** **8**A), while the drug resistance of TMEM52B‐P18 and TMEM52B‐P20 overexpressed NPC cells HONE‐1, HK‐1, and S18 to DDP was significantly enhanced (Figure 8B). We treated the cells with different concentrations of the Akt activation inhibitor LY294002, and the concentration of 20 µm significantly inhibited Akt phosphorylation (Figure 8C). When 20 µm LY294002 was used to treat NPC cells, the drug resistance curve shifted significantly to the left, indicating that LY294002 could significantly reverse the drug resistance induced by TMEM52B (Figure 8D). In the experiment of detecting subcutaneous tumor formation in mice with HONE‐1 cells (Figure 3G), we also detected the tumor formation ability of mice treated with DDP (Figure S3E, Supporting Information) (The sample of NS group in Figure S3E (Supporting Information) is the same as the sample in Figure 3G). The results showed that the tumor volume and the weight of tumor tissue decreased after DDP treatment. Although there was no significant difference compared with the normal saline (NS) treated group, the P value of tumor volume in LV‐Ctrl group (0.09) was still significantly lower than that of LV‐TMEM52B‐P18 (0.89) and LV‐TMEM52B‐P20 (0.54) (Figure S3F,G, Supporting Information), the P value of tumor weight in LV‐Ctrl group (0.12) was also significantly lower than that of LV‐TMEM52B‐P18 (0.37) and LV‐TMEM52B‐P20 (0.24) (Figure S3H, Supporting Information). These results all prove that TMEM‐52B‐P18 and TMEM52B‐P20 can make NPC cells resistant to cisplatin, and AKT phosphorylation inhibitors can reverse this effect. Meanwhile, AKT inhibitors can significantly weaken the promoting effects of TMEM52B‐P18 and TMEM52B‐P20 on cell migration and cell invasion (Figure 8E–H), the results show that the effect of TMEM52B is dependent on the activity of AKT. In addition, despite complete inhibition of TMEM52B‐P18's function, P20 still exhibits some functional activity which further confirms, as mentioned in our previous studies, that TMEM52B‐P20 can regulate cell metastasis by influencing the stability of E‐cadherin, in addition to activating AKT.

## 3 Discussion

In this study, we aimed to understand whether the novel tumor‐related gene TMEM52B can regulate the occurrence and development of NPC and the underlying molecular mechanism. To do so, we first implemented transcriptomic sequencing of normal nasopharyngeal cells and NPC cells, and identified TMEM52B as an up‐regulated gene of NPC by differential expression analysis. Given that we had previously shown that TMEM52B promotes the progression of esophageal squamous cell carcinoma (ESCC) by increasing the EMT of ESCC cells,^[^ ^21^ ^]^ we followed TMEM52B further as a potential Oncogenic factor in NPC.

After performing a series of in vitro and in vivo analyses, we provide evidence that TMEM52B facilitates AKT and EMT signaling that contributes to the oncogenesis and metastasis of NPC. More importantly, our work has now discovered a previously unknown difference in the oncogenic roles of the TMEM52B‐18 and TMEM52B‐P20 isoforms in NPC. Although both are important for activating AKT signaling and inducing EMT in NPC cells, and played comparable roles in promoting NPC growth, TMEM52B‐P20 had a significantly stronger effect than TMEM52B‐P18 in accelerating NPC metastasis. This finding implies that targeted therapy for TMEM52B‐P20 might be more effective in nasopharyngeal cancer patients with high metastatic ability.

As a recently discovered gene,^[^ ^11^ ^]^ TMEM52B function in cancer has not been studied intensively. Besides its oncogenic role in ESCC as shown by our earlier study,^[^ ^21^ ^]^ others have reported a tumor‐suppressing role of TMEM52B in colorectal cancer; TMEM52B interacts with E‐cadherin to restrict its cleavage into a soluble form, leading to the activation of EGFR and downstream suppression of MAPKs and AKT signaling.^[^ ^13^ ^]^ Studies to date have revealed that many genes known to have important roles as tumor suppressors are also implicated in tumorigenesis; examples include wild‐type p53 in hepatocellular carcinoma,^[^ ^22^ ^]^ phosphatase and tensin homolog in pre‐B acute lymphoblastic leukemia,^[^ ^23^ ^]^ p21 in high grade/poorly differentiated, advanced human carcinomas,^[^ ^24^ ^]^ and ATM in breast cancer.^[^ ^25^ ^]^ Therefore, the NPC‐promoting function of TMEM52B in our present study and its tumor‐inhibiting function in previous reports may reflect a context‐dependent manner by which TMEM52B exerts its influences in cancer.

In this study, we identified a dominant role for TMEM52B‐P20 in promoting migration, invasion, and metastasis of NPC cells, although TMEM52B‐P18 and TMEM52B‐P20 promoted NPC growth to a similar extent. Hence, we speculate that irrespective of whether TMEM52B functions as a tumor suppressor or an oncoprotein, TMEM52B‐P20 has a more consequential function.

Others have shown the different subcellular distributions of TMEM52B isoform 1 and isoform 2 — with the former found in the cytosol and cell membrane but the later at the cell membrane edge — in HEK293E cells.^[^ ^13^ ^]^ Here, we demonstrated the presence of both cytosolic and plasma membrane‐associated TMEM52B‐P20 but only cytosolic distribution of TMEM52B‐18 in HONE‐1 cells. We substantiated that the plasma membrane association of TMEM52B‐P20 distinguishes its role in NPC metastasis from TMEM52B‐P18, by generating TMEM52B‐P20 mutants deficient in membrane localization; unlike wild‐type TMEM52B‐P20, these mutants were not more effective than TMEM52B‐P18 in promoting the migration and invasion of HONE‐1 cells. Moreover, mutation also abolished the upregulated E‐cadherin ubiquitination observed with wild‐type TMEM52B‐P20. Given that the horizontal ubiquitination transition is very important for the stability and functional regulation of E‐cadherin, we conducted a preliminary validation of the known E‐cadherin‐associated E3 ubiquitin ligases and found that the interaction of NEDD4 and MDM2 with E‐cadherin can be promoted by TMEM52B‐P20. Meanwhile, NEDD4 could also be localized to cell membranes,^[^ ^26^ ^]^ which lead us to speculate that it may play a more important role in this regulatory mode. In this way, we confirmed that TMEM52B‐P20 regulates cellular function by influencing the interaction of E‐cadherin with NEDD4. These results explain that TMEM52B‐P20 has more advantages than TMEM52B‐P18 in the regulation of cell metastasis. We also demonstrated that NEDD4, MDM2, and RNF43 interact with TMEM52B‐P20 in the HONE‐1 cells (Figure S3A–C, Supporting Information), while Hakai does not (Figure S3D, Supporting Information). In summary, the change in E‐cadherin stability depends on the formation of a complex of TMEM52B‐P20, E3 ubiquitin ligase, and E‐cadherin, which sets the tone for further exploration of their interaction patterns. Based on the current study, we can not determine whether TMEM52B‐P20 has a specific enzyme activity, so we prefer TMEM52B‐P20 to play a bridge role in this complex, bridging the distance between E3 ubiquitin ligase and E‐cadherin.

Interestingly, we noticed that HONE‐1 cells overexpressing TMEM52B‐P20 had a marked difference in cellular morphology (Figure 4C), strongly suggesting that TMEM52B‐P20 promotes the motility and invasiveness of HONE‐1 cells via mechanisms other than EMT. This may be due to the fact that E‐cadherin is an important factor in maintaining cell adhesion. E‐cadherin acts as a bridge between the extracellular matrix and the cytoskeleton and lack of E‐cadherin in tumors results in changes in cell morphology.^[^ ^27^, ^28^ ^]^ Another plausible explanation is that filopodium‐like protrusions have also been recognized as functional structures that enable metastatic tumor cells to colonize secondary tissues or organs.^[^ ^29^, ^30^, ^31^ ^]^ Indeed, inhibiting fascin, the main actin‐bundling protein in filopodia, can impede tumor invasion and metastatic colonization.^[^ ^32^, ^33^, ^34^ ^]^ Furthermore, a previous study found a link formation consisting of vimentin intermediate filament‐plectin‐invadopodia‐filamentous actin in invasive bladder cancer cells, which could stabilize invadopodia and confer high metastatic capabilities to cancer cells.^[^ ^35^ ^]^ We contend that TMEM52B‐P20 may be involved in invadopodia formation through an interaction with cytoskeletal linker protein, for example, PLEC‐1 screened by mass spectrometry (Figure S1A, Supporting Information), which may explain the distinctive cellular morphology of TMEM52B‐P20 overexpressed HONE‐1 cells observed here. Our study had not been able to fully answer this question, as TMEM52B‐P18 also interacted with PLEC‐1, so more evidence about the role of TMEM52B‐P20 in invadopodia formation is now needed in order to investigate this hypothesis.

In terms of promoting NPC growth, we found that TMEM52B‐P18 and TMEM52B‐P20 have a comparable role, both in vitro and in vivo, which is significantly suppressed by PGK1 siRNA pretreatment. We also demonstrated that both TMEM52B‐P18 and TMEM52B‐P20 interact with PGK1, implying that the tumor growth‐promoting role of both isoforms depends largely on their association with PGK1. PGK1 is a well‐known oncoprotein reported to be involved in the activation of PI3K/AKT signaling.^[^ ^36^, ^37^, ^38^ ^]^ Increased expression of PGK1 can activate the PI3K/AKT signaling pathway in gastric cancer progression, leading to a poor prognosis.^[^ ^39^ ^]^ In our study, overexpression of either TMEM52B‐P18 or TMEM52B‐P20 failed to increase the levels of phosphorylated AKT in HONE‐1 cells with PGK1 knockdown, followed by failure to accelerate cell proliferation, suggesting that the similarity of TMEM52B isoforms in promoting NPC growth may lie in PGK1‐mediated AKT activation. PGK1is known to function not only as a glycolytic enzyme, but also as a protein kinase capable of intermolecular autophosphorylation.^[^ ^40^ ^]^ Our study did not elucidate the molecular mechanism by which TMEM52B isoforms together with PGK1 activate AKT signaling; whether the interaction between TMEM52B and PGK1 affects the protein level or the enzymatic activities of PGK1 needs further investigation.

Our current study confirmed at the cellular level that TMEM52B can increase the resistance of NPC cells to DDP. In vivo experiments, although there were certain differences between the DDP treated group and the control group, the P‐value was greater than 0.05. Further clarifying the role of TMEM52B in the regulation of cell resistance will be of great significance for future clinical applications and will also be the focus of our follow‐up research.

## 4 Conclusion 

In summary, we show that TMEM52B represents a promising biomarker for early diagnosis and prognostic prediction in NPC. Moreover, TMEM52B suppression may be a new strategy for treating NPC. Specifically, we show that TMEM52B regulates NPC through two pathways. Cytoplasmic TMEM52B‐P18 and TMEM52B‐P20 promote AKT phosphorylation by binding to PGK1, thereby activating a range of downstream cellular functions. On the other hand, membrane‐bound TMEM52B‐P20 affects the ubiquitination of E‐cadherin and degrades it, thus promoting metastasis. An interesting point to consider going forward is that different expression levels of different isoforms may correspond to different tumor stages and be a useful guide to patient prognosis and treatment. Studying functional differences between TMEM52B isoforms might, therefore, lead us to pay more attention to the concept of treating the same disease by different methods. Indeed, targeting different isoforms of the same gene and different sublocalizations of the same protein, will no doubt open up a new direction for personalized, precision medicine, which we anticipate will improve the accuracy of targeted therapy.

## 5 Experimental Section

### Cell Lines and Cultures

Normal NPEC lines (N2‐Bmi‐1, N5‐Tert, and NP69) were cultured in keratinocyte serum‐free medium (Gibco, USA) supplemented with human recombinant epidermal growth factor (ThermoFisher Scientific, USA), bovine pituitary extract (ThermoFisher Scientific, USA), and 1% penicillin/streptomycin (Gibco, USA). Human NPC cell lines (5‐8F, 6–10B, CNE‐1, CNE‐2 (S18), HK‐1, HNE‐1, HONE‐1, and SUNE‐1) were cultured in RPMI 1640 (Gibco, USA) supplemented with 10% fetal bovine serum (Gibco, USA) and 1% penicillin/streptomycin. These cell lines were incubated at 37 °C in a humidified chamber with 5% CO2. All NPEC and NPC cell lines were generously provided by Professor Musheng Zeng from the Sun Yat‐Sen University Cancer Center and Professor George Sai‐Wah Tsao (HK‐1 and HONE‐1) from the University of Hong Kong. All cell lines were authenticated using short‐tandem repeat profiling, and tested for mycoplasma contamination.

### Transfection of siRNA, shRNA Vectors, and Expression Vectors

For TMEM52B targeting, SUNE‐1 and S18 cells were transfected with siRNA control or TMEM52B siRNA sequences (5′‐GCAGAAAGCACCTGATCTA‐3′ and 5′‐GGGTACATCTCTGGTATATAT‐3′) (Shanghai GenePharma Co., Ltd., Shanghai, China) using Lipofectamine RNAiMAX transfection reagent (Invitrogen, USA) according to the manufacturer's instructions. The same TMEM52B siRNA sequences were used to construct shRNA vectors, GV493‐TMEM52B‐sh1 and GV493‐TMEM52B‐sh2 (5′‐GCAGAAAGCACCTGATCTA‐3′ and 5′‐GGGTACATCTCTGGTATATAT‐3′) by GeneChem (Shanghai, China). Two expression vectors, GV358‐TMEM52B‐P18 and GV358‐TMEM52B‐P20, were also constructed by GeneChem. All plasmids were verified by DNA sequencing. For TMEM52B overexpression, HONE‐1 and HK‐1 cells were transfected with control, TMEM52B‐P18, or TMEM52B‐P20 vectors using Lipofectamine 3000 (Invitrogen, USA) according to the manufacturer's instructions. HONE‐1 cells were transfected with PGK1 siRNA sequences (5′‐GCTTCTGGGAACAAGGTTA‐3′) (Shanghai GenePharma Co., Ltd., Shanghai, China) using Lipofectamine RNAiMAX transfection reagent (Invitrogen, USA) according to the manufacturer's instructions. To establish stable strains, SUNE‐1 and HONE‐1 cells were transduced with lentivirus packing shRNA or expression plasmids for 10 h in the presence of polybrene (5 µg mL^−1^) and were subsequently selected with puromycin (1 µg mL^−1^) for 1 week.

### RNA Isolation and Real‐Time PCR Analyses

Total RNA was extracted using TRIzol reagent (Invitrogen, USA), and cDNA was synthesized using reverse transcriptase (Invitrogen, USA). Real‐time PCR was performed using Power SYBR Green PCR Master Mix (Applied Biosystems) on the ABI 7300 Real‐Time PCR system (Applied Biosystems). Relative gene expression was calculated using the comparative threshold cycle (2^–ΔΔCT^) method. All reactions were performed in triplicate.

### RNA Sequencing and Data Analysis

Total RNA was isolated from cultured cells using TRIzol reagent (Invitrogen, USA). RNA sequencing was carried out by Shenzhen BGI Health Technology Co., Ltd. (Shenzhen, China) following standard protocols. Library sequencing was run on an BGISEQ‐500 System. Standard bioinformatics analysis was performed by Shenzhen BGI Health Technology Co., Ltd. RNA sequencing data reported in this paper were deposited in the Genome Sequence Archive of the National Genomics Data Center, China National Center for Bioinformation/Beijing Institute of Genomics, Chinese Academy of Sciences (GSA‐Human: HRA004364), and are publicly accessible at https://ngdc.cncb.ac.cn/gsa‐human. Gene expression data were quantified and normalized using BeadStudio software (Illumina). Genes with fold change ≥ 2 and a false discovery rate of < 0.01 were considered differentially expressed.

### Western Blotting

Cells were lysed and sonicated. Total protein concentrations were measured by a Pierce BCA Protein Assay Kit (ThermoFisher Scientific, USA). Western blotting was performed using the following antibodies: anti‐TMEM52B (NBP2‐49272, Novus), anti‐Flag (#14 793, Cell Signaling), anti‐Flag (F1804, Sigma‐Aldrich), anti‐N‐cadherin (sc‐7939, Santa Cruz Biotechnology), anti‐E‐cadherin (#3195, Cell Signaling), anti‐vimentin (sc‐7557, Santa Cruz Biotechnology), anti‐Twist (ab50887, Abcam), anti‐PGK1/2 (sc‐48342, Santa Cruz Biotechnology), anti‐PLEC‐1 (#12 254, Cell Signaling), anti‐phospho‐AKT (S473) (#4060, Cell Signaling), anti‐phospho‐AKT (T308) (#4056, Cell Signaling), anti‐AKT (pan) (#4691, Cell Signaling), and anti‐β‐tubulin (ab6046, Abcam). Conjugated horseradish peroxidase‐was used as the secondary antibody. Proteins were detected using an enhanced chemiluminescence kit (Millipore, USA) and visualized with the GeneGnome XRQ Chemical Imaging System (Gene Company Limited, Hong Kong, China).

### Immunoprecipitation and Mass Spectrometry

Stable strains of HONE‐1 were cultured and lysed in a weak RIPA buffer (Beyotime Biotechnology, China) containing 1% NP‐40 and 0.25% deoxycholate supplemented with protease inhibitor (Bimake, USA). Cell lysates were incubated with Anti‐FLAG M2 magnetic beads (Sigma‐Aldrich, USA) at 4 °C overnight. Magnetic beads were washed and proteins were eluted by boiling in sodium dodecyl sulfate sample buffer, then subjected to western blotting using the antibodies indicated, or to sodium dodecyl sulfate (SDS) polyacrylamide gel electrophoresis followed by Coomassie Brilliant Blue staining. Specific bands that were only observed in lysates from cells overexpressing TMEM52B isoforms were excised and subjected to mass spectrometry.

### In Vivo Ubiquitination Assay

HA‐Ub, Flag‐E‐cadherin, and TMEM52B related plasmids were overexpressed in HEK293 cells. The cells were lysed 48 h later with IP buffer (1% NP‐40 and cocktail) containing 0.5% SDS, and pre‐denatured at 95 °C for 10 min, then ultrasonically disintegrated. The sample was centrifuged at 4 °C for 10 min, then Flag M2 beads were used to concentrate the supernatant and associated proteins were detected by western blotting.

### Immunohistochemistry

Human NPC tissue microarray was purchased from Shanghai Outdo Biotech Co. Ltd (HNasN132Su01; Shanghai, China). Immunohistochemical staining was carried out as previously reported.^[^ ^41^ ^]^ The sections were incubated with anti‐TMEM52B (205013‐T08, Sino Biological), anti‐Ki67 (ab15580, Abcam), or anti‐PCNA (sc56, Santa Cruz Biotechnology) primary antibodies, followed by biotin‐conjugated goat anti‐rabbit or biotin‐conjugated goat anti‐mouse secondary antibodies (MXB Biotechnologies, China). The proportion of stained cells (<5% = 0; 5–25% = 1; 26–50% = 2; 51–75% = 3; >75% = 4) and staining intensity (negative = 0; weak = 1; moderate = 2; strong = 3) were independently scored by three pathologists and the average value from the three pathologists was used as the final score.

### Immunocytochemistry

NPC cells grown on cover slides were fixed with 4% paraformaldehyde for 15 min, then permeabilized with 0.1% Triton X‐100 in PBS for 20 min, underwent blockage with 3% bovine serum albumin in PBST for 1 h, and were immediately incubated with anti‐E‐cadherin (#EP700Y, Abcam) antibodies overnight at 4 °C. The nuclei were stained with DAPI (#D1306, Thermo Fisher). Fluorescent images were captured with a confocal fluorescent microscope (ZEISS, Germany).

### Cell Proliferation, Colony Formation and 5‐ethynyl‐2′‐deoxyuridine Incorporation Assays

Cell viability was determined using the cell counting kit‐8 (CCK‐8; Dojindo, Japan) according to the manufacturer's instructions. Briefly, the cells were incubated with CCK‐8 reagent for 2 h at 37 °C at different time points as indicated, and the absorbance at 450 nm was measured using a microplate reader (Bio‐Rad Laboratories, Inc.). The colony formation assay was done by seeding 1000 cells per well into six‐well plates and culturing the cells under routine conditions for 10–15 days. Cell colonies were fixed with 4% paraformaldehyde and stained with 0.1% crystal violet (Beyotime Biotechnology, China); colonies were photographed and counted. Cell proliferation was evaluated with the 5‐ethynyl‐2′‐deoxyuridine (EdU) incorporation assay. Briefly, cells were incubated with 10 µm EdU for 2 h and stained with Apollo fluorescent dye (Cell‐Light EdU Apollo567 In vitro Kit, Ribobio, China) according to the manufacturer's instructions. Images were captured under a regular fluorescent microscope (Olympus IX73, Germany).

### Transwell Migration and Invasion Assays

Cell migration and invasion abilities were measured with 8 µm pore transwell chambers (BD Biosciences, USA) that were coated without or with Matrigel (BD Biosciences, USA). Medium supplemented with 10% fetal bovine serum was placed in the lower chambers. NPC cells (5 × 10^4^ for migration and 8 × 10^4^ for invasion) prepared in serum‐free medium were seeded in the upper chambers. After incubation at 37 °C for 24 h, migrating or invading cells were fixed with 4% paraformaldehyde, stained with 0.1% crystal violet. The number of migrated or invaded cells was calculated using five random fields photographed under a light microscope (Nikon DS‐Ri2, Tokyo, Japan).

### In Vivo Xenograft Tumor Models

Male BALB/c nude mice (4–5 weeks, 16–18 g) were purchased from Guangdong Medical Animal Experimental Center. All animal experiments were carried out in accordance with the principles and procedures outlined by the Experimental Animal Welfare Ethics Committee of the Shenzhen Peking University Hong Kong University of Science and Technology Medical Center. For subcutaneous tumorigenicity assay, 1 × 10^6^ SUNE‐1 or HONE‐1 cells pre‐infected with different lentiviral vectors were subcutaneously inoculated into the flank of BALB/c nude mice (n ≥ 5 per group). Once tumors were established, the mice were treated daily by oral gavage with either physiological saline or DDP(20 mg kg^−1^). The body weight and tumor size were measured every 4 days. The tumor volume was calculated as 0.5 × (length × width^2^). Fifteen days after injection, the mice were sacrificed and the tumors were excised for analysis. In the lung metastasis model, 5 × 10^4^ HONE‐1 cells were injected into mice via their tail vein. The body weight and physical condition were evaluated every 3 days. The mice were euthanized once the body weight had rapidly decreased to <15 g or their physical condition reached the humane endpoint, and the lungs were enucleated and embedded in paraffin for further analysis.

### Statistical Analysis

SPSS v.18.0 or GraphPad Prism 8.0 software were used for statistical analyses. Unpaired two‐tailed Student's t test were used to analyze Edu, colony, migration, and invasion of cells or tumor weight. The two‐way ANOVA test followed by Sidak multiple comparisons were used to analyze body weight and tumor size of nude mice or CCK8 of cells. A one‐way ANOVA test followed by Turkey's multiple comparisons was used to analyze TNM stage of NPC patients. The χ^2^ test was used to analyze the relationship between TMEM52B expression and various clinicopathologic characteristics. Receiver operating curve analysis showing area under the curve and 95% confidence interval (CI) was performed to assess the potential of TMEM52B to differentiate high‐risk from low‐risk NPC patients. The Kaplan‐Meier method was used to estimate survival curves, and the log‐rank test was used to compare them. For univariate and multivariate analyses, the Cox proportional hazard regression model was used to determine the effect of clinicopathologic variables and TMEM52B expression on survival. Data were represented as mean ± SD or mean ± SEM. A *p <* 0.05 was considered statistically significant.*p < 0.05, ** p < 0.01, *** p < 0.001, and ns means no significance.

## Conflict of Interest

The authors declare no conflict of interest.

## Author Contributions

Y.Z., Y.L., C.X., and Y.H. contributed equally to this work. Y.Z., Y.L., C.X., X.X., and Z.L. conceived and designed the study; Y.Z., Y.L., Y.H., Z.Y., T.W., L.M., X.L., W.Y., S.L., W.Z., F.Z., K.L., Y.Z., S.M., and Y.D. performed experiments in vitro and in vivo. G.N., X.F., and Z.W. provided reagents and materials; Y.L., C.X., and Y.Z. performed the statistical analyses. C.X., Y.Z., X.X., and Z.L. drafted and revised the manuscript. All authors contributed to data interpretation. All authors read and approved the final version of the manuscript.

## Supporting information

Supporting Information

Supplemental Data1

Supplemental Data2

Supplemental Data3

## Data Availability

The data that support the findings of this study are available from the corresponding author upon reasonable request.
