# Supplementary material for: TMEM52B Isoforms P18 and P20 Differentially Promote the Oncogenesis and Metastasis of Nasopharyngeal Carcinoma
Source: Adv Sci (Weinh). 2024 Jun 28;11(33):2402457. doi: 10.1002/advs.202402457 (PMC11434218; doi:10.1002/advs.202402457)
Supplement: Supplementary file 1 — Supporting Information [file ADVS-11-2402457-s002.pdf]

ADVANCED SCIENCE
Open Access

Supporting Information

for *Adv. Sci.*, DOI 10.1002/advs.202402457

TMEM52B Isoforms P18 and P20 Differentially Promote the Oncogenesis and Metastasis of Nasopharyngeal Carcinoma

Yuqi Zhu, Yanxin Lu, Chunhua Xu, Yuqian Huang, Ziyi Yu, Tongyu Wang, Longyi Mao, Ximian Liao, Shi Li, Wanqing Zhang, Feng Zhou, Kaiqing Liu, Yu Zhang, Wei Yang, Shasha Min, Yaqin Deng, Zaixing Wang, Xiaoqin Fan, Guohui Nie, Xina Xie and Zesong Li**

Supporting Information

TMEM52B isoforms P18 and P20 differentially promote the oncogenesis and metastasis of nasopharyngeal carcinoma

Yuqi Zhu[1,2,3†], Yanxin Lu[1,4†], Chunhua Xu[1,2†], Yuqian Huang[1†], Ziyi Yu[1], Tongyu Wang[1], Longyi Mao[1], Ximian Liao[1], Shi Li[1], Wanqing Zhang[1], Feng Zhou[5], Kaiqing Liu[1], Yu Zhang[1,2], Wei Yang[1,2], Shasha Min[1,4], Yaqin Deng[1], Zaixing Wang[6], Xiaoqin Fan[7], Guohui Nie[7], Xina Xie[1], Zesong Li[1,3,4,8*]*

† Yuqi Zhu, Yanxin Lu, Chunhua Xu, and Yuqian Huang contributed equally to this work.

***Corresponding author.**

E-mail: lzssc@email.szu.edu.cn; xiexina0204@email.szu.edu.cn

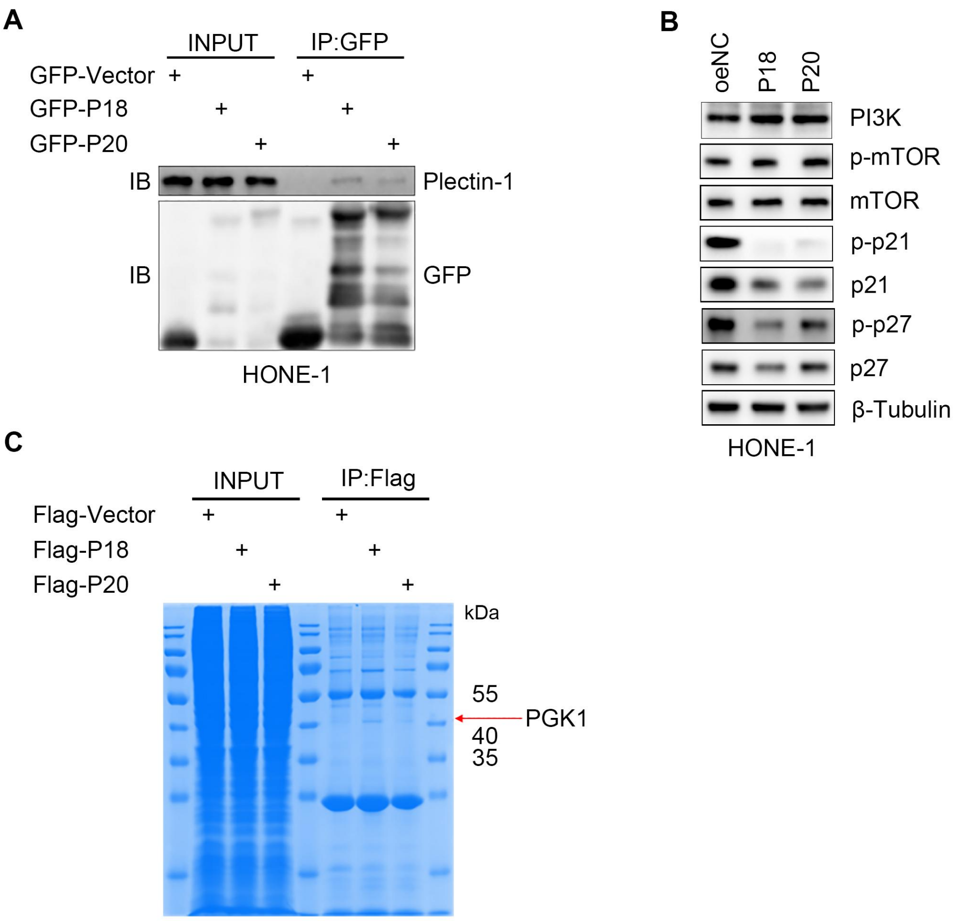

Figure S1. **A.** Cell lysates from HONE-1 cells transfected with control, TMEM52B-P18 or TMEM52B-P20 plasmids were immunoprecipitated with anti-GFP to pull down TMEM52B-P18 and TMEM52B-P20, followed by western blotting with anti-Plectin-1 or anti-GFP antibody on the precipitates and lysates, as indicated. **B.** Western blotting to determine the influence of TMEM52B-P18 and TMEM52B-P20 on the protein levels of PI3K, p-mTOR, mTOR, p-p21, p21, p-p27 and p27 in HONE-1 cells. **C**. Cell lysates from HONE-1 cells were immunoprecipitated with anti-Flag to pull down TMEM52B-P18 and TMEM52B-P20. The precipitates were analyzed by SDS-PAGE with Coomassie Brilliant Blue staining.

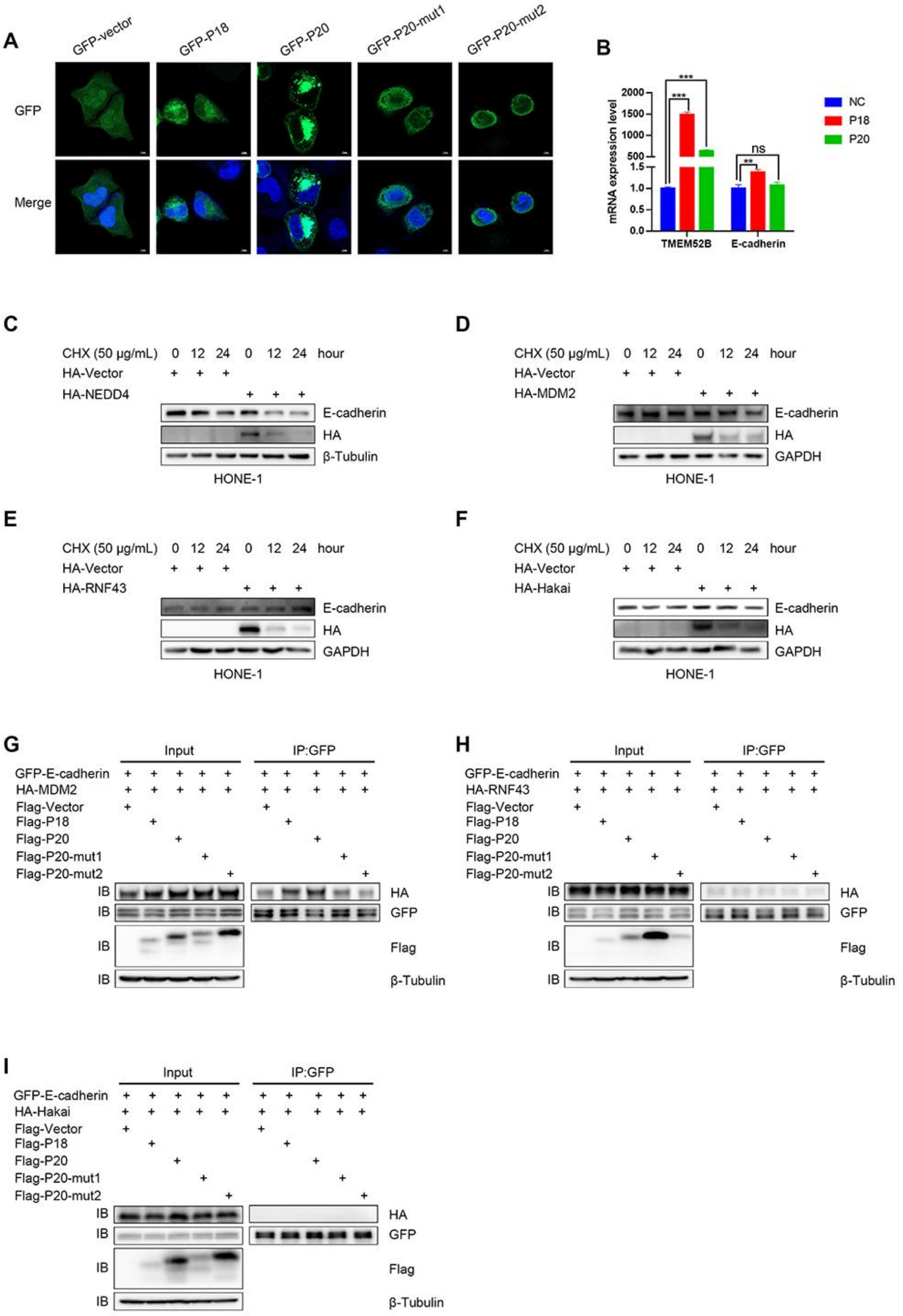

Figure S2. **A.** Localization of GFP-P18, GFP-P20 and GFP-P20 mutants by immunostaining analysis. Scale bar, 5 μm. **B.** Real-time PCR analysis of mRNA levels of E-cadherin and TMEM52B in HONE-1 cells overexpressing TMEM52B isoforms, β-actin was used as the internal control. **C-F.** HONE-1 cells were transfected with control or related E3 ubiquitin ligase

expression plasmids, followed by the treated with CHX. Western blotting was performed to estimate the E-cadherin with β-Tubulin as the loading control. **G-I.** Cell lysates from HONE-1 cells transfected with GFP-E-cadherin, TMEM52B and E3 ubiquitin ligase plasmids were immunoprecipitated with anti-GFP to pull down HA-MDM2, HA-RNF43 or HA-Hakai followed by western blotting with anti-Flag, anti-GFP or anti-HA antibody on the precipitates and lysates, as indicated. Data were represented as mean ± SD , n=3, $**p < 0.01$, $***p < 0.001$, ns means no significance. P value calculated by unpaired two-tailed Student's t test .

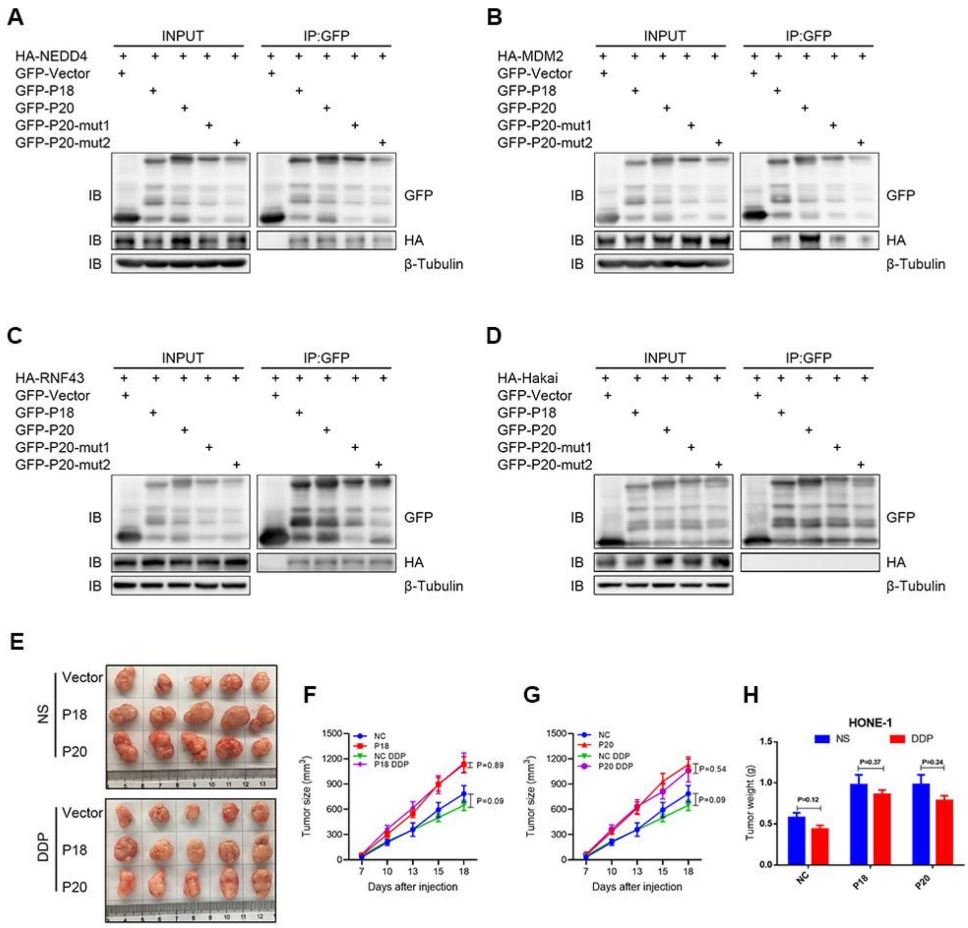

Figure S3. A-D. Cell lysates from HONE-1 cells transfected with TMEM52B and E3 ubiquitin ligase plasmids were immunoprecipitated with anti-GFP to pull down HA-NEDD4, HA-MDM2, HA-RNF43 or HA-Hakai followed by western blotting with anti-GFP or anti-HA antibody on the precipitates and lysates, as indicated.**E-H.** HONE-1 cells pre-infected with control, TMEM52B-P18-expressing, or TMEM52B-P20-expressing lentivirus were subcutaneously inoculated into the flank of each BALB/c nude mouse (n=5 per group). Once tumors were established, the mice were treated daily by oral gavage with either physiological saline or DDP. DDP was given at (20 mg kg^{-1}). The tumor photographs (E), growth curves of tumors (F, G) and tumor weights (H) are shown. Data were represented as mean ± SEM , n=5. P value calculated by two-way ANOVA .
